# Between Heaven and Hell: Experiences of Preoperative Pain and Pain Management among Older Patients with Hip Fracture

**DOI:** 10.1177/23779608221097450

**Published:** 2022-05-06

**Authors:** Anna Unneby, Yngve Gustafson, Birgitta Olofsson, Britt-Marie Lindgren

**Affiliations:** 1 Department of Nursing and Department of Surgical and Perioperative Science Orthopedics, 375281 Umeå University, Umeå, Sweden; 2 Department of Community Medicine and Rehabilitation, Geriatric Medicine, 375281 Umeå University, Umeå, Sweden; 3 Department of Nursing, 375281 Umeå University, Umeå, Sweden

**Keywords:** femoral nerve block, hip fracture, opioids, pain, pain managment, peripheral nerve block

## Abstract

**Introduction:**

Among older adults, hip fracture is a common and serious consequence of a fall. Preoperative pain is common and often severe among patients with hip fracture. Opioids are usually used but have many side effects. One alternative is a femoral nerve block, which has been shown to reduce pain and lower the need for opioids. However, to our knowledge no study has explored qualitatively how patients with hip fracture experience treatment with femoral nerve block.

**Objective:**

The aim of this study was to explore experiences of preoperative pain and pain management among older patients with hip fracture who had received a femoral nerve block.

**Method:**

A qualitative design with semi-structured interviews (n = 23) conducted 2–6 days after surgery. Inclusion criteria were Swedish-speaking patients aged 70 years or older with hip fracture admitted to the orthopedic ward, treated with femoral nerve block before nursing actions. Data were analyzed with qualitative content analysis.

**Results:**

Our result revealed one theme, hovering between heaven and hell, with five subthemes: how the pain was described – no pain, to worst pain and everything in between; they were dealing with pain in their own way; felt dependent on staff´s willingness to relieve pain; pain management could be lifesaving and a near-death experience; and how they experienced memory loss with respect to the pain and pain management.

**Conclusion:**

The experience of pain and pain management was described as hovering between heaven and hell. We conclude that independent of which pain management given, staff should have an individualized pain mangement approach towards the patient in order to achieve well managed pain.

## Introduction

Hip fracture causes severe complications due to fragility and comorbidity resulting in high mortality and morbidity (Flikweert et al., 2018; Katsoulis et al., 2017; Ranhoff et al., 2010; Unneby et al., 2020). Sweden has among the highest incidence of hip fractures in the world (Kanis et al., 2012). Women are overrepresented (66%) and have a higher mean age (83 years) compared to men (81 years) (RIKSHOFT, 2021).

This older population having physiological changes and increased number of comorbidities presents a challenge in both assessing and treating pain (Elsevier & Cannada, 2020; Wennberg et al., 2018). Opioids are usually used to treat pain among patients with hip fracture (Bollinger et al., 2015; NICE, 2017). However, side effects of opioids are common (Daoust et al., 2020) and have been described by patients to be worse than the pain itself (Hallström et al., 2000). An alternative to opioid pain management is the femoral nerve block (FNB), which has shown to reduce preoperative pain according to pain score and lower the amount of preoperative opioids needed, a result also seen among those with dementia disorders (Unneby et al., 2017).

Research reports that patients experience the waiting time to surgery as long, with feelings of hunger, thirst and dry mouth, leading them to fear they will suffer complications (Hommel et al., 2012). The preoperative phase has been described as an overall feeling of fuzziness accompanied by fear and pain (Olsson et al., 2006). After the hip fracture surgery, recalling the preoperative pain and the inability to move the leg made patients believe they might never walk again (Olsson et al., 2006). The pain has been emphasized by patients with hip fracture to be constant, severe and most intense preoperatively (Hall-Lord et al., 2004; Hestdal & Skorpen, 2019). Further, patients reported that the preoperative pain caused feelings of suffering, anxiety and stress (Hall-Lord et al., 2004; Hestdal & Skorpen, 2019; Hommel et al., 2012). Pain is important to manage, as it can have severe consequences such as compromised pulmonary and cardiac function, delirium, depression, and sleep and appetite disturbances (Maher et al., 2012; Sanzone, 2016).

There are few studies focusing on patients’ experiences of preoperative pain and pain management. Further, to our knowledge there is no study focusing on the experiences of receiving and being treated with an FNB as an alternative to pain management in the preoperative phase due to a hip fracture. The aim of this study was to explore experiences of preoperative pain and pain management among older patients with hip fracture who had received an FNB.

## Method

### Context/Setting

The study was conducted in one orthopedic ward (OW) and one geriatric ward (GW), specialized in rehabilitation among orthopedic patients. In the preoperative phase all participants were treated at the OW. By routine, patients with cervical hip fracture are treated at the GW postoperatively, while those with a trochanteric fracture are treated at the OW postoperatively. However, some of the patients with a trochanteric fracture who are in need of further rehabilitation are transferred after consultation to the GW. Regarding preoperative pain management in the care chain when suffering a hip fracture, patients may receive analgesics in the ambulance, at the emergency department (ED) and at the OW. By routine, long-lasting opioids and paracetamol are given at specific times, and intravenous and oral short-acting opioids are given when necessary; these are the primary analgesics given. FNB is a standard pain management and can be given at the ED or at the OW. Pain management in this manuscript is an overall term and defined as opioid use (intravenous/pills) and/or FNB.

### Participants

A purposive sample of patients with hip fracture who could talk about their experiences of preoperative pain and pain management were invited to participate in this study. Patients were eligible for inclusion if they met the following criteria: 70 years of age or older, Swedish-speaking patients with hip fracture (cervical or trochanteric) admitted preoperatively to the OW and treated with FNB before nursing actions, that is, before nursing actions involving movement of the patient, for example, before mandatory showering on a stretcher. Patients were recruited during the first postoperative day up to one week postoperatively. The first author received information about all eligible patients with hip fracture, determined whether they met the inclusion criteria and then invited the patients to participate in the study. The first author then asked the patients who met the inclusion criteria if they wanted to participate in the study. All participants eligible for inclusion were given verbal and written information about the study by the first author. Those who agreed signed a consent form, and a time, chosen by the patient, was set for interview. Background data such as sex, age, living condition, type of fracture, status according to the ASA-physical status classification system (Owens et al., 1978), and place and time for FNB were registered from medical records.

In this study, 23 participants were included, 19 women and 4 men. Median age was 78 years (range = 72–94 years). Thirteen were diagnosed with a cervical fracture and 10 with trochanteric fracture. Nineteen participants were living independently, with or without home care service, and four at a residential care facility. Sixteen participants had an ASA grade 3–4. The FNB was given at the ED to 7 participants, and 16 received FNB at the OW. All participants except one received intravenous opioids somewhere during the prehospital care chain (pre-hospital, ED or OW). Thirteen participants received intravenous opioids at the ED and nine participants received intravenous opioids at the OW.

### Data Collection

Semi-structured interviews were used for data collection (Brinkmann & Kvale, 2014). The first author conducted all interviews except two. Those two interviews were conducted by a trained PhD colleague. The interviews were performed in private in the patient's room or in an examination room expedition on the orthopedic or geriatric ward, depending on where the patient was cared for postoperative. The interviews were conducted between December 2017 and September 2019. The median time for performing the interviews was at postoperative day 5 (range = 2–6 days). The interview guide consisted of questions such as ‘Can you tell me about the pain relief you received before your surgery?’ and ‘Can you tell me about the injection you received in your groin/the FNB?’ During the interview follow-up and clarifying questions, ‘Can you tell me more about that?’ and ‘Please elaborate on what you mean by that’ were used. The interviews lasted between 7 and 40 min (median = 25 min).

### Data Analysis

Qualitative content analysis was used to analyse the interviews (Graneheim et al., 2017; Graneheim & Lundman, 2004; Lindgren et al., 2020). All interviews were conducted before the analysis started. The first author read the transcribed interviews several times and listened to the audio recordings to become familiar with the data and to understand and get a sense of the whole. To ensure trustworthiness, one of the co-authors read all of them, and the two others did read five each of the transcribed interviews. The transcripts were imported into MAXQDA qualitative data management software to systemically sort and code the data (Kuckartz & Radiker, 2019). Next, the first author started the process of decontextualisation by dividing the interview texts into meaning units (words, sentences or paragraphs related to the same central meaning). Initially, meaning units were separated in two domains, that is, pain or pain management. Thereafter, the meaning units were condensed and labeled with codes describing the manifest content. Before the next step in the analysis, all authors took part in the coding before sorting them regarding similarities and differences. An example of the analysis process is that codes such as ‘had no choice’, ‘forced to take it and not to protest’, ‘staff took care of everything’, and ‘to be in safe hands’ were interpreted and sorted into the subtheme dependent on staff willingness to relieve pain

In the recontextualisation after codes were sorted, we needed to take a step back and bring the two domains together, since they had fulfilled their purpose. All authors were involved in the process of abstraction and interpretation, which led to the final wording and agreement on subthemes and theme. The theme revealed in this study should be interpreted as a metaphor (Graneheim et al., 2017).

### Ethical Considerations

All participants were informed that their participation was voluntary and that they could withdraw from participation at any time without consequences. The authors were not involved in the treatment of the participants included in the study. Interviewing patients regarding their experiences about pain and pain management can evoke memories, which can include unpleasant experiences. Questions can be experienced as too personal or probing. Further, interviewing patients soon after their hip fracture may be strenuous for them. However, the experience of having someone who is genuinely interested in hearing about their experiences can be a relief (Gaydos, 2005).

## Results

Participants’ descriptions of their experiences of pain and pain management in the preoperative phase revealed one theme with five subthemes. Table 1 shows an overview of the results.

**Table 1.** Overview of Subthemes and Theme Revealed in the Analysis among Participants Descriptions of Their Experiences of Pain and Pain Management in the Preoperative Phase.

Subthemes	Theme
No pain, to worst pain and everything in between	Hovering between heaven and hell
Dealing with pain in their own way	
Dependent on staff's willingness to relieve pain	
Lifesaving or a near-death experience	
Experiencing memory loss	

### Hovering Between Heaven and Hell

Participants were hovering between heaven and hell in the preoperative phase regarding pain and pain management. Participants described how they experienced pain as ranging from no pain to worst pain and everything in between. They described how they dealt with their pain in their own way but also felt dependent on staff's willingness to relieve pain, and that the pain management could be lifesaving but also like a near-death experience. Some participants reported that due to memory loss they had difficulty remembering the pain as well as the pain management they had received.

### No Pain, to Worst Pain and Everything in Between

Some participants stated that they were surprised about the diagnosis of serious injury, as in a hip fracture, since the pain was not noticeable, and some described how they sometimes did not feel any pain in rest in the preoperative phase. As one participant expressed it,

Well, it went well, I didn’t feel so much, so I didn’t think it was so serious, but it was probably more serious, but I did not experience it so. (P 10)

Some participants also recalled that they did not feel any difference in pain after receiving pain relief, since they did not feel any pain. Some participants reported that the mandatory surgical shower and other nursing activities in the preoperative phase, which implicates a need of movements and transfers, went well, without having pain from the broken hip.

In contrast to those experiencing no pain, other participants described the preoperative pain as ‘horrible’, ‘unbearable’, ‘infernal’ and hopelessly severe, without relating to any pain management. They expressed how the pain was so severe that they lacked words and that the pain from the hip fracture was so terrible that it was something they did not want to experience again. The severity of existing pain led some of the participants to assume that the hip was broken, before they had been x-rayed.

Moving the leg by oneself or with help was described by the participants as the worst. One participant expressed it like this:

Patient: At the ward it was so damn painful, so I didn’t want to, I almost didn't dare to, move…

Interviewer: What happened if you tried to move or if the staff tried to move you? How was the pain then?

Patient: Excruciating. I didn't want anyone to touch me. (P 6)

The pain was described as so severe that it was impossible to get undressed, even if staff helped them. It was further related that staff needed to cut the clothes apart to get them off. Participants expressed how they felt anxious before nursing actions that included lifting the leg because of the severe pain that movements caused them. On the one hand, some participants expressed how the pain increased so much in movement that it was described as a near-death experience, and on the other hand, some participants expressed a feeling of relief when the staff did not move them because the pain was so severe at the slightest movement. Some participants described the surgery shower on the stretcher as the worst experience regarding pain. As one participant said:

The pain was worse when I needed to shower, it was so painful, I thought I was going to die when I needed to move myself. (P 4)

Some participants described the pain from the hip fracture as manageable, without connecting it to any pain management. On the one hand, some participants described experiencing pain at first but the pain decreasing over time, regardless of pain management received. On the other hand, some participants described the pain from the hip fracture as variable, but also said the pain was the same, regardless of pain management received. Some participants described how the pain was present, but even with a hip fracture they could still stand on the leg. Yet others expressed that difficulty moving the leg was a bigger problem than the pain.

### Dealing with Pain in Their own way

Participants expressed some individual strategies to deal with the pain that the hip fracture caused them. They described how the pain faded away through their own thoughts, that they could distract themselves by thinking about other things, where others also described that they dealt with their pain by screaming, where some participants expressed movements of the leg to be worst. Screaming loudly was one way to handle the pain during movements, as a way to let off steam. One participant said:

It becomes something unconscious, a way to react, I think, I don't know, or yes, it is a way to react. You get busy in some way when you scream. (P 23)

Even though having a hip fracture was described as painful, some participants also described how they decided to tolerate the pain and accept the pain in general, including during nursing actions. As one participant said,

You need to tolerate the staff working with you, or else they let you be. I mean, they cannot do magic tricks either, they need to help you shower. They are careful. I must say that the staff are amazing, because you cannot just complain and whine if you have hurt yourself, and I mean the staff are not to blame for me having hurt myself. (P 10)

Some participants had their own ideas, based on advice from others, on how to manage pain on their own. Advices such as having a pillow or similar under the knee, which also resulted in a feeling that the pillow was the best pain relief.

### Dependent on Staff's Willingness to Relieve Pain

The participants stated that staff had an important role for patients to achieve well-managed pain. They felt that staff invested a lot of time in the patients to achieve well-managed pain and they felt that staff had a goal of ensuring that patients were pain-free before surgery. Some of the participants reported receiving pain relief before the x-ray was performed to confirm a hip fracture. Some participants describe that aids, such as sliding boards during transfers, were important, because they made the transfer less painful. Further, some participants described the staff also putting something under the knee and how that sometimes was the only thing that decreased the pain. The participants also noted that the staff took care of everything, which made them feel calm; they trusted the staff, and they were able to completely surrender to them. As one participant said:

I think that the actual care, the whole procedure, I came into safe hands. I was taken care of and it felt very nice. (P 16)

The participants expressed feeling safe when the anesthesiologist performing the FNB demonstrated knowledge of the technique, as occurred when the anesthesiologist was perceived as prepared and explained the procedure to them. Overall, receiving the FNB was regarded by the participants as ‘not a big thing’. One participant said:

The anaesthesiologist told me everything he did … and the anaesthesiologist had an assistant who was introduced, just like a training or something. [The interviewer asks a probing question]. I thought that this anaesthesiologist knows what he is doing … it made me pretty calm. (P 8)

In difference from participants describing satisfying pain management abilities, other participants described unsatisfying experiences of staff's pain management abilities. They described how staff administered pain relief almost all the time without success, despite all that pain management. Further, they described how staff only ran around to give pain relief, with no time for other things. The participants described that the staff did not discuss alternatives or options for pain management with them, that it was already decided over their heads. They tried to speak up about their unwillingness to receive opioids and pills, but it was to no avail; they described how staff gave opioids despite the protests from participants. As one participant stated:

It was morphine, paracetamol and God knows what, and I asked which pills it was because I didńt recognise them, but I was forced to take them, and there was no time to protest. (P 1)

Further, some participants expressed frustration due to the staff's difficulties inserting a venous cannula when receiving intravenous opioids and how that delayed the possibility of receiving pain relief. Participants described feeling frustrated when the anesthesiologist did not show any interest in or focus on the participants before and during the administration of the FNB. As one participant said:

And then I thought it would be great if the pain would go away. She was nice; there was nothing wrong with that, but she talked on the phone and perform the block simultaneously, and I do not know how many phone calls she called during the time she performed the block…. And why I react is because a driver cannot talk on the phone when he drives the car. (P 12)

Some of the participants also described unsatisfying experiences when staff tried to decrease pain by laying the leg in a comfortable way using pillows or similar but failed. No matter how the staff tried to help the participants to change position, it did not decrease the pain.

### Lifesaving or a Near-Death Experience

Participants reported that the analgesic effect from pain management itself was the reason why they experienced a satisfying pain situation. They described how the pain released when they arrived both at the ED and at the OW after receiving pain relief. Further, participants described how the pain management overall was helpful; however, some described that thinking about everything else in the preoperative phase made it hard to remember in detail how the pain felt or how it changed when they received pain management.

Some participants said that the amount of intravenous opioid given was a lifesaver and that opioids made the pain fade away. Intravenous opioids were described as necessary before transfers between bed and surgical stretcher or other similar nursing actions. Further, some expressed that intravenous opioids were a better choice for pain management compared to pills. For example, they made it possible to have a calm night without being disturbed, since it is less disruptive to receive intravenous medication than to swallow pills, lying in bed with a hip fracture. As one participant expressed it:

I have received a lot of pain relief, and it is thanks to that I think I have survived. Oh my God, I have had such horrible pain, so that's the way it is. (P 6)

Some participants expressed how the pain suddenly changed and disappeared after receiving the FNB. They described how they felt relaxed and relied on the analgesic effect after receiving the FNB. Some participants described how the leg became anaesthetised with retained sensation, how they did not have any pain when the staff moved the leg or other nursing actions. The participants who needed an additional FNB said that they then understood what an FNB was intended to do. Participants commented as follows:

One thing is for sure, you don't resist, you trust the block that the anaesthesiologist performed. (P 8)

I think it was after the block, I experienced that there wasn't so much pain anymore, because it is so terribly awful to have so much pain. (P 9)

Contrasted with a feeling of well-managed pain, some other participants described how both oral and intravenous opioids were given without decreasing pain. They expressed how opioids made them feel intoxicated and declared that opioids were the reason for their memory loss. Furthermore, participants attributed symptoms such as nausea, drowsiness and dizziness to the opioids. When receiving intravenous opioids, it was essential to receive medicines for nausea as well. Participants declared that opioids made them experience nightmares and hallucinations, and that it led to anxiety about death and feelings of fighting for their own life. As one participant said:

Patient: It was awful, I got pain medicine and I got such horrible nightmares [crying desperately].

Interviewer: Do you still remember these nightmares even today?

Patient: Yes, I remember them; in the last nightmare I came in to a hospital, a small room or what it's called, and there was a pram that was a winter pram, which I did not think it would be. It should be summer, and I thought, oh my God, no. I’m sitting on and suffocating the little baby [crying desperately]. (P 21)

Contrary to the block being an effective pain relief management both in rest and in movements, some other participants expressed that the FNB did not make any difference to the pain and that they had not understood that FNB was intended for pain management. When the FNB did not reduce pain as intended, staff told the participants that it could be because of differences in how well the FNB decreases pain, and when the FNB did not decrease pain, they were offered a repeated FNB.

Even though the participants could not remember in detail which pain management they had received, they still proposed limiting the opioids or at least offering some milder treatment, saying that they would rather experience more pain than accept opioids. Further, participants stated that there are advantages and disadvantages with opioids, but overall, they are not harmless. Participants emphasized that an FNB could be an alternative to receiving less opioids, especially if opioids cause unwanted side effects. As one participant said:

It made a big difference with the block, pain became lighter, and then I think it's good if you don't need quite as much morphine, if the morphine makes you feel nauseous. (P 23)

### Experiencing Memory Loss

Although this study focuses on experiences of pain and pain management among patients with hip fracture, an important finding was that some participants expressed problems remembering the preoperative phase. They described how everything had become a fog and were confused when they tried to think back to the preoperative phase. Some participants described how they had an apathetic feeling in the preoperative phase and how that was the reason why they experienced memory loss. Even when the participants expressed difficulties in remembering whether they had received some pain management preoperatively, they came to the conclusion that they most likely did. Another factor mentioned was that many things were happening around them, making it hard to keep different aspects separated. Participants also likened the preoperative phase to how it might feel to be dead. When they were asked specific questions about the FNB, they had difficulty remembering the procedure and the analgesic effect.

## Discussion

Participants described their experiences of pain and its management in the preoperative phase as a feeling of hovering between heaven and hell. They described a range of pain from no pain, to worst pain and everything in between, and how they dealt with pain in their own ways. They also felt dependent on staff´s willingness to relieve pain and that pain management could be lifesaving but also a near-death experience. Some participants had difficulties recalling how they experienced pain and received pain management, due to memory loss.

The hip fracture forced the participants in the present study to interrupt their everyday lives. Other researchers have reported that patients described hip fracture as life changing, with feelings of being hopeless and helpless (Archibald, 2003; Gesar et al., 2017). Some participants in the present study described how, despite the life-changing situation, they felt they were in safe hands, being calm and trustful and having no pain and/or well-managed pain during the hospital stay. This was interpreted in the present study as being in heaven. In difference, when participants described pain as the worst, comparing the preoperative phase to being dead and feeling anxiety about death, not being listened to, not being involved in the pain management despite protests, and further, when participants narrated in detail the experience of hallucinating and how that made them cry desperately, this was interpreted as being in hell.

A systematic review by Abrahamsen and Nørgaard (2020) about elderly patients’ perspectives on treatment, care and rehabilitation after a hip fracture, reports that the preoperative pain experienced by patients with hip fracture was severe and intense. That is somewhat contradictory to the present results, where some participants reported that they did not experience pain in the preoperative phase while other participants expressed how pain in movement was the worst, and they preferred not to move the leg on their own or have staff move the leg – they preferred no movements at all. In clinical care and for patient safety, a preoperative routine is a mandatory surgical shower. This could be problematic knowing that some participants expressed pain as very difficult while others did not. The results in the present study can be seen as a help towards staff understanding the difficulty facilitating an adequate pain management for older patients with hip fracture.

Similar to our results, other studies report that patients described the pain as being worst in movements, with the severity of pain causing patients to avoid moving both preoperatively and postoperatively (Hallström et al., 2000; Hommel et al., 2012). In our results, some participants described the pain in movement as a near-death experience and said that movements and nursing actions caused anxiety. Similar to our results, one study reported that patients described pain and their inability to move causing and increasing feelings of suffering (Hestdal & Skorpen, 2019).

In the present study, some participants described ways of dealing with the pain on their own or needing to tolerate the pain. Similar results are echoed in a study by Hallström et al. (2000) where patients stated that the pain was a natural result of the injury and that they had to tolerate it. How patients experience and deal with pain in different ways, even when the pain management is similar, might be related to the complexity and subjectivity of pain (Melzack, 2005; Younger et al., 2009).

Our results showed that the interaction between staff and participants was important in achieving well-managed pain, regardless of the pain relief they received. Participants felt on one hand calm and safe if staff were present and responsive. Similar results are shown in other studies, where patients felt satisfied when staff created a secure feeling (Hommel et al., 2012). Having patients involved in their own care has shown not only to reduce feelings of stress and anxiety but also to increase satisfaction and motivation regarding the care received (Sahlsten et al., 2008).

Our results also showed on the other hand that some other participants did not feel seen and listened to, nor were they allowed to be involved in decisions regarding their pain management. Similar was reported by Malmgren et al. (2014) where patients with hip fracture described how they tried to be more involved in decisions about additional pain management but were informed that this was not possible. An earlier study by Hallström et al. (2000) showed that communication between patients and staff about pain was unclear. Patients did not want to bother the staff and therefore did not ask for help, or they saw staff as experts and thought that they were being given all pain treatment possible. However, nurses in the same study said that they expected patients to ask for analgesics (Hallström et al., 2000).

Staff not being present and responsive to the patient's views regarding pain relief indicates that they did not see the person behind the patient. An important approach in person-centred care is to see patients as human beings with reason, will, feelings and needs as a way to involve them as active partners in their own care and treatment (Ekman et al., 2011). Our results indicate a lack of person-centred pain management. Person-centred pain management was previously described by Avallin et al. (2018) among patients with abdominal pain, across the acute care chain. To achieve person-centred pain management, it is essential to establish and maintain trust, communication and information about pain and pain management together with use of well-evaluated analgesics. Trust could be established even during a short time of interaction, which made patients feel comfortable dealing with their pain (Avallin et al. 2018).

Regardless of the interaction with staff, the analgesic effect from pain medication itself was reported by some of the participants to be the reason that well-managed pain was achieved. Similar results were described in a study by Hestdal and Skorpen (2019), where pain management was expressed by patients to be important. Patients from other studies expressed, on one hand, fear of unwanted side effects and of being overdosed by staff, but on the other hand, also trust in the staff's knowledge about the medicine they were being offered (Hommel et al., 2012) and not being afraid of side effects (Hestdal & Skorpen 2019).

In our study pain medication was described as a reason for well managed pain, while other participants expressed that pain relief did not decrease pain and that opioids created nightmares, hallucinations and anxiety about death, leading them to refuse pain relief. That is similar to other studies where unwanted side effects such as hallucinations were ascribed to pain management, which caused patients to refuse pain relief and to describe the side effects as being worse than the pain (Hallström et al., 2000; Hommel et al., 2012). In a study by Olsson et al. (2007) about effects of nursing interventions within an integrated care pathway for patients with hip fracture pain relief was considered important, and nurses were told to pay special attention to whether patients experienced inadequate pain relief.

In a study by Henningsen et al. (2018) about patients’ experience of peripheral nerve blocks when undergoing ankle surgery, participants described the value of retaining mental alertness and ability to move, and how participants expressed the block to be an effective pain management. This is similar to our results, where participants stated that FNB made the pain disappear and made it possible for staff to move the leg without increasing the pain. Our results showed that some of the participants had difficulty remembering receiving a block and the effect on pain thereafter. Similar results are reported in a qualitative study illuminating whether a fascia iliaca compartment block is acceptable to patients with hip fracture (Evans et al., 2019). In our study, some of the participants were uncertain about FNB as a pain management technique. Similar was found in the study by Henningsen et al. (2018) where some patients had difficulty understanding the effect and course with the nerve block. In our study, participants felt secure and calm when the FNB was explained thoroughly and when staff were perceived as present. This can be compared with a study by Malmgren et al. (2014) where patients responded positively and felt respected when they were offered a choice between general or spinal anaesthesia during surgery. Henningsen et al. (2018) stated that patients who receive peripheral nerve blocks should receive thorough and repeated information, as it could increase the overall clinical usefulness of nerve blocks.

In the present study, some participants experienced memory loss during the waiting time to surgery. They described both an overall dimness thinking back, and also regarding specific questions about pain and pain management. Some participants described how they felt apathetic in the preoperative phase and also compared the preoperative phase as being dead. Hommel et al. (2012) reported that patients with hip fractures experienced loss of perception of time and place. Further, a study by Aronsson et al. (2014) focusing on experience from the pre-hospital emergency care among patients with suspected hip fracture reported that the patients experienced confusion or memory loss even while in the pre-hospital context.

There could be several different reasons why they experienced difficulty recalling the preoperative phase. The experienced memory loss might be due to receiving opioids pre- and postoperatively (Chau et al., 2008), which also was one explanation given by the participants in the present study. Or, that with aging, the risk for cognitive decline, including memory dysfunction, increases and gets worse after hospitalization and surgery (Terrando et al., 2011). Further, Unneby et al. (2020) reported that elderly patients with hip fractures often suffer from pre- and postoperative delirium. Patients’ memories of pain have been investigated in several studies, and a systematic review by Adamczyck et al. (2019) reported that recalling pain can be precise, but also overestimated and underestimated. We can only speculate whether some of this might explain why the participants in the present study had difficulties remembering something from the preoperative phase.

### Methodological Considerations

Some methodological considerations need to be discussed. The research group considered and discussed before the study started when the interviews should take place. Since the interview guide also involved questions regarding pain and pain management in the postoperative phase, it was necessary to perform the interviews postoperatively. The timing of the interviews with respect to recalling the preoperative phase was discussed in particular, as the preoperative phase and the FNB are a small part of the hospital stay, and it was expected that if the interviews were conducted a long time after the preoperative phase the risk of inaccurate recall would increase.

In this study a purposive sample was used, and as research reports, women are overrepresented among patients with hip fractures (RIKSHOFT, 2021), which is in line with the distribution of women and men in our study.

Some interviews can be considered short, however, Sandelowski (1995) argue that whether data are sufficient is not dependent of the number of participants nor of the length of for example interviews. Instead, it is a matter of experience in assessing the quality of the data in relation to the aim of the research. In our study data from the interviews were rich and varied in content and were therefore assessed as suitable for analysis.

The results are a co-creation not only between the researchers and the participants but also between the researchers and the text (Graneheim et al., 2017). Working in a research group with a variety of understandings, different insights and contexts is an advantage, since it can prevent the researcher's preunderstandings from influencing the results. One of the researchers lacked experience in the context of OW and patients with hip fracture but contributed with extensive qualitative methodological experience.

### Implications for Practice

Patients with hip fracture describes pain differently in the preoperative phase, from no pain, to worst pain and everything in between. Independent of which pain management given to the patient, staff should have an individualized pain mangement approach towards the patient in order to achieve well managed pain.

## Conclusion

Pain and pain management among older patients with hip fracture were experienced as hovering between heaven and hell. Staff with responsibility for evidence-based pain management should always take into account the unique patient cared for and that person's expectations. The next step would be to investigate how staff experience caring for patients with hip fracture and treated with FNB.

## Ethics

This study was approved by a regional ethical review board in Sweden (DNR 2016/387-31).
